# Quality of Life Assessment for Tonsillar Infections and Their Treatment

**DOI:** 10.3390/medicina58050589

**Published:** 2022-04-25

**Authors:** Berit Hackenberg, Matthias Büttner, Michelle Schöndorf, Sebastian Strieth, Wendelin Schramm, Christoph Matthias, Haralampos Gouveris

**Affiliations:** 1 Department of Otorhinolaryngology, Head and Neck Surgery, University Medical Center Mainz, 55131 Mainz, Germany; michelleschoendorf@gmail.com (M.S.); christoph.matthias@unimedizin-mainz.de (C.M.); haralampos.gouveris@unimedizin-mainz.de (H.G.); 2 Institute of Medical Biostatistics, Epidemiology and Informatics (IMBEI), University Medical Center Mainz, 55131 Mainz, Germany; matbuett@uni-mainz.de; 3 Department of Otorhinolaryngology, University Medical Center Bonn (UKB), 53127 Bonn, Germany; sebastian.strieth@ukbonn.de; 4 GECKO Institute for Medicine, Informatics and Economics, Heilbronn University, 74081 Heilbronn, Germany; wendelin.schramm@hs-heilbronn.de

**Keywords:** tonsillitis, peritonsillar abscess, tonsillectomy, utility, quality of life, hospital stay, 15D questionnaire

## Abstract

*Background and Objectives:* Tonsillar infections are a common reason to see a physician and lead to a reduction in the patients’ health-related quality of life (HRQoL). HRQoL may be an important criterion in decision science and should be taken into account when deciding when to perform tonsillectomy, especially for chronic tonsillitis. The aim of this study was to determine the health utility for different states of tonsillar infections. *Materials and Methods:* Hospitalized patients with acute tonsillitis or a peritonsillar abscess were asked about their HRQoL with the 15D questionnaire. Patients who had undergone tonsillectomy were reassessed six months postoperatively. *Results:* In total, 65 patients participated in the study. The health states of acute tonsillitis and peritonsillar abscess had both a utility of 0.72. Six months after tonsillectomy, the mean health utility was 0.95. *Conclusions:* Our study confirms a substantial reduction in utility due to tonsillar infections. Tonsillectomy significantly improves the utility and therefore HRQoL six months after surgery.

## 1. Introduction

Acute tonsillitis is an infectious inflammation of the palatine tonsils. It can be caused by a wide spectrum of viruses and bacteria [1]. The diagnosis is usually based on clinical findings and it may occur in all age groups, with a peak in school-aged children [1,2]. There are no reliable data on the prevalence of recurrent acute tonsillitis in Germany. However, acute tonsillitis is one of the most common reasons to see a physician, and Kvestad et al. report a lifetime prevalence of 11.7% in Norway [3,4].

It remains unclear when tonsillectomy should be performed as opposed to standard conservative treatment, i.e., watchful waiting with/without analgesics or antibiotic treatment [5]. Nevertheless, in cases of chronic or recurrent tonsillitis, tonsillectomy has proven to be an important intervention to improve the patient’s health-related quality of life (HRQoL) [6,7]. These patients do not only suffer from tonsil-related symptoms. They also report more healthcare visits and more days absent from school or work [7]. This broader reduction in HRQoL must be taken into account when making decisions on how to treat these patients. While costs are often easily comparable within the same healthcare system, HRQoL as a clinical outcome measure may be difficult to compare between studies. In order to achieve comparability of results even between different diseases and interventions, a common outcome measure must be found. For this purpose, the quality-adjusted life year (QALY) has become the gold standard in cost–utility analysis [8]. It takes into account the quality of life that a patient attributes to each year of life lived with a disease [9]. This is done by multiplying a utility value for the health state by the time spent in that state. The utility value is rated on an interval scale between 0 (equivalent to death) and 1 (perfect health) [8]. Several generic questionnaires have been validated to determine utility values. One of them is the 15D questionnaire. It is a 15-dimensional generic questionnaire for self-administration that has been translated into German and other languages [10]. Due to its design, it produces a single index number on a scale from 0 to 1 and can thus be used to calculate QALYs. It was published in 1981 according to the WHO definition of health and revised in 1986 and 1992. Because of the vast accumulated experience with its use during the last few decades, it has been used to assess various health conditions for common otolaryngologic diseases [11,12,13,14]. This allows for good comparability in this area of research.

The aim of this study was to determine utility values for different health states of (peri-)tonsillar infections.

## 2. Material and Methods

### 2.1. Study Design

This prospective study was conducted at the Department of Otorhinolaryngology at the University Medical Center Mainz, Germany between June 2018 and October 2020. The study cohort consisted of hospitalized patients who presented in the emergency department with acute tonsillitis or peritonsillar abscess and patients scheduled for a planned tonsillectomy for chronic tonsillitis. Inclusion was based on ICD-10 codes specified for the admission diagnosis and obtained from the hospital’s electronic medical record (EMR). The following ICD-10 codes were used: J03.0, J03.8 and J03.9 for acute tonsillitis; J36 for peritonsillar abscess; and J35.0, J35.8 and J35.9 for chronic tonsillitis. Exclusion criteria were insufficient knowledge of the German language and age < 18 years. The study was approved by the Institutional Review Board (Ethics Commission of the State Chamber of Physicians of Rhineland-Palatine, number: 837.464.17 (11298)). Written informed consent was obtained from all study participants.

### 2.2. Data Acquisition

Age, sex and smoking status (yes/no) were obtained from the EMR of the participants. The Charlson Comorbidity Index (CCI) was completed based on the EMR. The CCI was developed to classify comorbidities to predict mortality risk and has been widely used to control for comorbidities in clinical studies [15,16]. It includes 19 comorbid conditions. Conditions with a worse prognosis, such as metastatic tumors, are weighted higher. Therefore, the scale ranges from 0 to a maximum of 37 points.

In addition, the 15D questionnaire (see below) was given to participants during their hospital stay or mailed 6 months postoperatively in the case of tonsillectomy. If the questionnaire was not returned within 4 weeks, one reminder was sent by mail, e-mail or through telephone contact, depending on which form of contact the participant had chosen when informed consent to study participation was obtained.

The 15D questionnaire aims at assessing the patients’ HRQoL in 15 dimensions [17]. These dimensions are: Mobility, Vision, Hearing, Breathing, Sleeping, Eating, Speech, Excretion, Usual activities, Mental function, Discomfort and symptoms, Depression, Distress, Vitality and Sexual activity. In each dimension, respondents rated their current health status on a 5-point ordinal scale. In this study, the instrument was used as a single index to obtain a single number between 0 and 1 representing total HRQoL, where 0 equals death and 1 means perfect health. The minimal important change (MIC) for the 15D scores was valued to be 0.015 [18].

**Figure 1:**
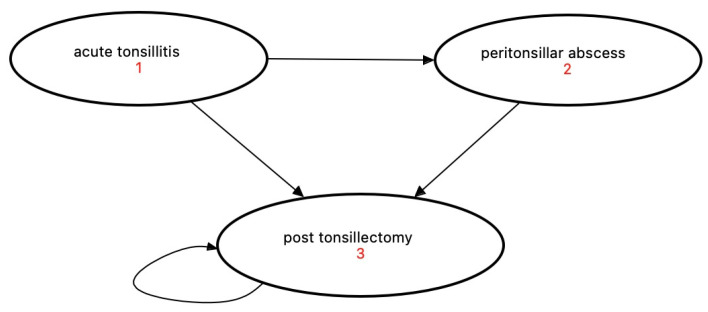
Health states of (peri-)tonsillar infections: (1) acute tonsillitis, (2) peritonsillar abscess and (3) post tonsillectomy.

Health states were defined as acute tonsillitis (health state 1) for acute infection, peritonsillar abscess (health state 2) for a complication and post-tonsillectomy (health state 3) for the participants’ condition 6 months after surgery (Figure 1). Although there is controversy regarding the pathogenesis of peritonsillar abscess in the literature, peritonsillar abscess is used in this study as a clinical case of a complication of (peri-)tonsillar infection [19].

Patient characteristics are expressed as mean or median values or percentages according to the data. Univariate comparisons between the three health states were conducted using the Chi-Square or Kruskal–Wallis test depending on the data.

## 3. Results

**Figure 2:**
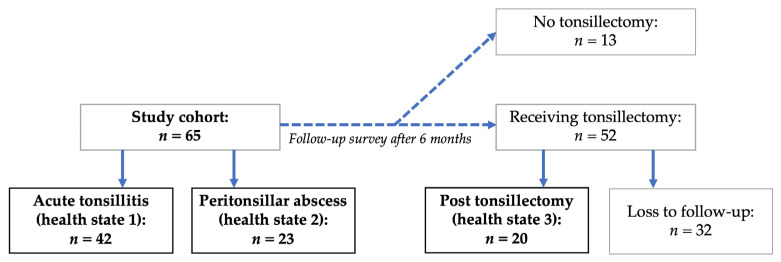
Participant inclusion.

A total of 65 participants could be included. Overall, 42 patients were included in health state 1 and 23 patients in health state 2. Of these 65 participants, 52 underwent tonsillectomy and were contacted by either phone or mail (depending on their choice) again six months postoperatively. With a response rate of 38.5%, 20 participants answered the questionnaire six months postoperatively. Their responses six months postoperatively were summarized as health state 3. See Figure 2 for the inclusion process.

Table 1 contains the descriptive statistics of all health states. For health state 1, one participant reported one comorbidity (congestive heart failure) and one participant reported two comorbidities (liver disease, diabetes mellitus). In health state 2, one participant reported one comorbidity (myocardial infarction). The participant with two comorbidities from health state 1 was included in health state 3 six months later and reported the same two comorbidities.

**Figure 3:**
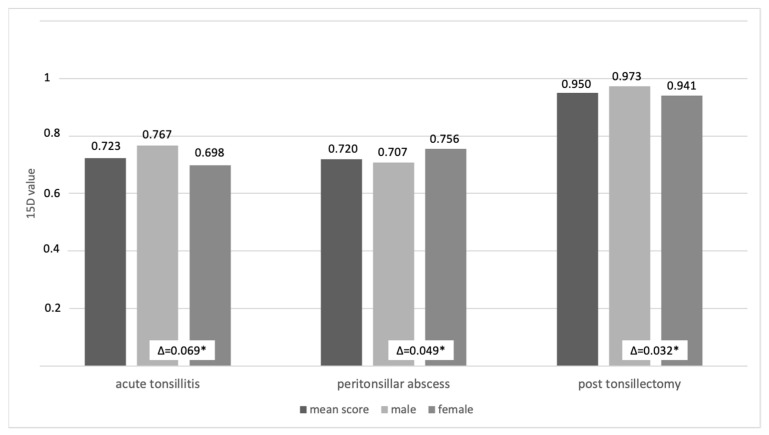
Mean utility scores by health state and gender. * = differences (Δ) above minimal important change of 0.015 (Alanne).

Health state 1 (acute tonsillitis) and 2 (peritonsillar abscess) both had a mean health utility of 0.72, while health state 3 (post tonsillectomy) had a mean health utility of 0.95 (see Table 2). In health state 1 and 3, males reported a higher mean utility than females. In health state 2, females reported a higher mean utility than males (see Figure 3).

**Figure 4:**
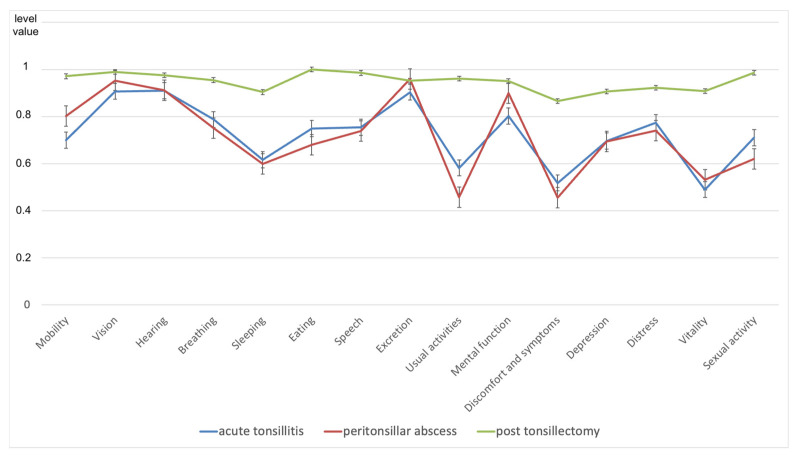
The 15D results of health state 1–3 for all 15 dimensions; results are expressed as mean + SEM (standard error of mean).

Health state 1 showed the lowest value on the Vitality item and the highest on the Hearing item. Both health states 2 and 3 showed their minimal values for the item Discomfort and symptoms, while health state 2 had its maximum for item Vision and health state 3 for the item Eating (see Figure 4).

For the difference between health state 1 and health state 3 (Δ1;3), all items were above the threshold of 0.015 for the minimum important change (see Table 3). The mean Δ1;3 was 0.22 (standard deviation: 0.11), with a maximum Δ1;3 of 0.42 for the item Vitality and a minimum Δ1;3 of 0.05 for the item Excretion. From health state 2 to health state 3 (Δ2;3), all items except Excretion were above the threshold of 0.015, with a mean Δ2;3 of 0.23 (standard deviation: 0.15). The maximum of Δ2;3 was 0.50 for Usual activities and the minimum of Δ2;3 was 0.04 for Vision.

## 4. Discussion

Health state utility values represent important criteria for clinical decision makers. Therefore, health state utility values are a fundamental requirement for future decision analyses. With this prospective study, we provide utility values for the clinical case of tonsillar infection, its complication (abscess) and its treatment.

We found that hospitalized patients with acute tonsillitis and peritonsillar abscess reported the same health utility of 0.72. This health utility increased significantly to 0.95 six months after tonsillectomy. Tonsillectomy can therefore be considered an important tool for improving patients’ HRQoL. This effect was strongest for limitations in sleep, usual activities, discomfort and vitality.

The 15D questionnaire used in this study is a single index instrument based on multiattribute utility theory. It determines a value on a scale from 0 to 1, where 0 is equivalent to death and 1 is equivalent to full health. Therefore, its results are not interpreted in absolute categories, but rather should be discussed in relation to other values or in terms of how they change over time or as a result of an intervention. This will be done in the following.

Our study confirms a relevant decrease in quality of life due to tonsillitis and its complications. This finding can be even better appreciated when comparing the utility values of our patients with those found in other significant medical conditions. For instance, newly diagnosed individuals with diabetes in the United Kingdom Prospective Trial have shown even higher quality of life metrics than our patients have, with an average value of 0.78 [20]. Patients with severe COPD in a home-based disease management program in Germany have reported a health utility of 0.76 [21]. Additionally, with a range of 0.65 to 0.88 (depending on severity), patients with depression have also reported a mean utility of 0.76 [22].

The change in utility between acute tonsillitis/peritonsillar abscess and the status after tonsillectomy was above the minimum important change (MIC) of 0.015 as stated by Alanne et al. [18]. They used a subjective five-level global assessment scale as an external anchor and included 4903 hospitalized patients in their study. Of these, 168 patients had tonsillar problems requiring tonsillectomy and were interviewed at baseline and 6 months postoperatively. These characteristics compare well with our study cohort. Therefore, this MIC can be considered clinically relevant to hospitalized patients with tonsillar infections and their treatment. However, taking the aforementioned MIC value (namely, 0.015) as a threshold for significant change, tonsillectomy has been found to significantly improve HRQoL in vision and hearing in our cohort. Since there is no further literature on this topic, the clinical relevance of the aforementioned MIC value should be questioned at this point.

Several authors have investigated the impact of tonsillectomy on patients´ HRQoL in the past. Two studies used the Glasgow Benefit Inventory (GBI) to compare the patients´ HRQoL 12 months before and 12 months after tonsillectomy [7,23]. Both found that tonsillectomy significantly improved HRQoL. The GBI is an instrument commonly used in otolaryngology to measure the change in HRQoL due to surgical and non-surgical interventions [24]. Ericsson et al. used the Short Form-36 (SF-36) and the EuroQol Visual Analogue Scale to compare HRQoL before and after tonsillectomy and tonsillotomy [25]. Both interventions improved HRQoL. A shorter version of the SF-36, the SF-12 Health Survey, has been used in another study along with a disease-specific instrument, the Tonsil and Adenoid Health Status Instrument (TAHSI) [26]. While the disease-specific instrument showed an improvement in HRQoL in all dimensions, the SF-12 showed a significant change only in the physical functioning subscale.

The 15D questionnaire was used by Wiksten et al. to measure changes in HRQoL before and after tonsillectomy [11]. A cohort of 124 participants included patients with chronic tonsillitis, enlarged tonsils causing snoring or swallowing problems and recurrent tonsillitis or abscess. The authors found no significant differences in HRQoL among these groups, which is consistent with the results of our study. The items Breathing, Sleeping, Discomfort and symptoms and Vitality were significantly impaired in their cohort before surgery. Nonetheless, the reported overall utility before tonsillectomy was 0.939 in their study, a significantly higher value than the one found in our study. This may be due to the fact that the largest patient group in their baseline cohort had only enlarged tonsils without any infectious focus. There were only a few (n = 13) participants with recurrent acute tonsillitis or abscesses in their study. The HRQoL 6 months after tonsillectomy was 0.956, which is in line with our results. Although their total baseline cohort was larger than ours, it appears that—based on our present findings—any acute tonsillar inflammatory state (including its complications, e.g., abscess) causes a significant decrease in HRQoL. Therefore, it may be suggested that chronic, non-inflammatory tonsillar conditions are associated with much higher utility values than inflammatory conditions are.

This study contains limitations. First, only hospitalized patients were included. Therefore, it may be assumed that these patients were more severely affected than patients who are treated as outpatients or by their primary care physician. The values obtained in this study may be used keeping this limitation in mind; this may involve, for example, studies examining the cost–utility of in-patient treatments. In addition, the cohort size is rather small, especially for the postoperative health state. On the other hand, the range of the confidence intervals of the resulting values is rather narrow and hence supports the plausibility of these results (Table 2).

In addition, the groups differed significantly in their gender and age distribution, with the peritonsillar abscess health state sample being slightly older and including more male participants. There is evidence that age may influence 15D questionnaire scores, with a slight tendency for a decrease in scores associated with increasing age [27]. Based on our cohort, which had a rather narrow age distribution (mean ages ranging between 25.4 years and 34.7 years), the potential decreases in HRQoL due to age are expected to be small. Gender differences are very specific according to the disease studied. Changes in HRQoL outcomes after tonsillectomy may considerably vary depending on the study cohort and the questionnaire used. In surveys using the GBI or the Adult Tonsil Outcome Inventory, gender had no effect on HRQoL outcomes [28,29]. On the other hand, Plath et al. found that female gender resulted in higher socioeconomic item subscores when using the Tonsillectomy Outcome Inventory 14 [30]. Other subscores were not affected by gender. The difference in mean utility between both genders was above the minimal important change of 0.015 and was therefore considered to be significant. With more male participants, the health utility in the peritonsillar abscess health state might be underestimated. To facilitate interpretation of these results, we reported health utility as mean scores and separately by gender.

With a relatively low return rate of 38.5%, it cannot be excluded that patients who had a more positive outcome after tonsillectomy were more likely to have answered the questionnaire. However, the results are consistent with previous literature reports and confirm the positive effect of tonsillectomy on the HRQoL of patients with recurrent tonsillitis. In addition, we controlled for the possible influence of comorbidities affecting HRQoL by applying the CCI. As shown in Table 1, the mean value of the CCI was below 0.1 in all groups. This suggests that our study participants were otherwise healthy, which can be explained by the young average age of our study cohort.

## 5. Conclusions

Hospitalized patients with acute tonsillitis and peritonsillar abscess reported a health utility of 0.72. Six months post-tonsillectomy, this health utility increased significantly to 0.95. Thus, this study confirms that tonsillitis and its complications lower a patient’s health utility. Tonsillectomy is a particularly effective means of improving health utility for limitations in sleep, usual activities, discomfort and vitality.

The health utility values reported in this study may inform future models of cost–utility analysis aiming at better depicting the benefits of the surgical versus non-surgical treatment of tonsillitis. This may be particularly relevant for recurrent acute disease states that significantly affect the patients in the mid- or in the long term.

## Figures and Tables

**Table 1:** Descriptive statistics of major epidemiologic features of the study participants.

Characteristic	Total (*n* = 65)	Acute Tonsillitis (*n* = 42)	Peritonsillar Abscess (*n* = 23)	Post-Tonsillectomy (*n* = 20) Follow-Up Survey	*p*-Value
age (years)					
**mean (SD)**	28.7 (9.7)	27.0 (7.5)	34.7 (12.3)	25.4 (7.6)	0.015 *
gender					
**male**	38 (44.7%)	15 (35.7%)	17 (73.9%)	6 (30.0%)	0.004 *
**female**	47 (55.3%)	27 (64.3%)	6 (26.1%)	14 (70.0%)	
smoking status					
**smoker**	33 (38.8%)	17 (40.5%)	11 (47.8%)	5 (25.0%)	0.29
**non-smoker**	52 (61.2%)	25 (59.5%)	12 (52.2%)	15 (75.0%)	
CCI					
**mean (SD)**	0.07 (0.33)	0.07 (0.34)	0.04 (0.21)	0.1 (0.44)	0.99

CCI = Charlson Comorbidity Index; * values represent significance with *p* < 0.05.

**Table 2:** Health utility values for tonsillitis-related health states.

Health State	Mean	SD	95% CI
**Acute tonsillitis**	0.72	0.16	[0.67; 0.77]
**Peritonsillar abscess**	0.72	0.15	[0.66; 0.78]
**Post-tonsillectomy**	0.95	0.07	[0.92; 1.00]

SD = standard deviation, CI = confidence interval.

**Table 3:** Change in health utility after tonsillectomy.

**Dimension**	**Mobility**	**Vision**	**Hearing**	**Breathing**	**Sleeping**	**Eating**	**Speech**	**Excretion**
Δ1;3	0.27	0.08	0.07	0.17	0.29	0.25	0.23	0.05
Δ2;3	0.17	0.04	0.06	0.20	0.31	0.32	0.25	−0.01 *
**Dimension**	**Usual** **Activities**	**Mental Function**	**Discomfort and Symptoms**	**Depression**	**Distress**	**Vitality**	**Sexual** **Activity**	
Δ1;3	0.38	0.15	0.35	0.21	0.15	0.42	0.28	
Δ2;3	0.50	0.05	0.41	0.21	0.18	0.38	0.37	

Δ1;3 = change in health utility from acute tonsillitis to post-tonsillectomy; Δ2;3 = change in health utility from peritonsillar abscess to post-tonsillectomy; * item below threshold of 0.015 for the minimal important change.

## Data Availability

Not applicable.
